# Oocyte activation and phospholipase C zeta (PLCζ): diagnostic and therapeutic implications for assisted reproductive technology

**DOI:** 10.1186/1478-811X-10-12

**Published:** 2012-07-09

**Authors:** Walaa M Ramadan, Junaid Kashir, Celine Jones, Kevin Coward

**Affiliations:** 1 Nuffield Department of Obstetrics and Gynaecology, University of Oxford, Level 3, Women’s Centre, John Radcliffe Hospital, Headington, Oxford, OX3, 9DU, UK

**Keywords:** Oocyte activation, Phospholipase c zeta (PLCzeta), Sperm, Male infertility, Assisted reproductive technology (ART), Therapeutic, Diagnostic, Biomarker

## Abstract

Infertility affects one in seven couples globally and has recently been classified as a disease by the World Health Organisation (WHO). While *in-vitro* fertilisation (IVF) offers effective treatment for many infertile couples, cases exhibiting severe male infertility (19–57%) often remain difficult, if not impossible to treat. In such cases, intracytoplasmic sperm injection (ICSI), a technique in which a single sperm is microinjected into the oocyte, is implemented. However, 1–5% of ICSI cycles still fail to fertilise, affecting over 1000 couples per year in the UK alone. Pregnancy and delivery rates for IVF and ICSI rarely exceed 30% and 23% respectively. It is therefore imperative that Assisted Reproductive Technology (ART) protocols are constantly modified by associated research programmes, in order to provide patients with the best chances of conception. Prior to fertilisation, mature oocytes are arrested in the metaphase stage of the second meiotic division (MII), which must be alleviated to allow the cell cycle, and subsequent embryogenesis, to proceed. Alleviation occurs through a series of concurrent events, collectively termed ‘*oocyte activation*’. In mammals, oocytes are activated by a series of intracellular calcium (Ca^2+^) oscillations following gamete fusion. Recent evidence implicates a sperm-specific phospholipase C, PLCzeta (PLCζ), introduced into the oocyte following membrane fusion as the factor responsible. This review summarises our current understanding of oocyte activation failure in human males, and describes recent advances in our knowledge linking certain cases of male infertility with defects in PLCζ expression and activity. Systematic literature searches were performed using PubMed and the ISI-Web of Knowledge. Databases compiled by the United Nations and World Health Organisation databases (UNWHO), and the Human Fertilization and Embryology Authority (HFEA) were also scrutinised. It is clear that PLCζ plays a fundamental role in the activation of mammalian oocytes, and that genetic, molecular, or biochemical perturbation of this key enzyme is strongly linked to human infertility where oocyte activation is deficient. Consequently, there is significant scope for our understanding of PLCζ to be translated to the ART clinic, both as a novel therapeutic agent with which to rescue oocyte activation deficiency (OAD), or as a prognostic/diagnostic biomarker of oocyte activation ability in target sperm samples.

## Introduction

Infertility affects one in seven couples globally [1-3], a worrying statistic which has led to the classification of infertility as a recognised disease by the World Health Organisation (WHO; [4]). In the UK, 1.5% of all births are the result of assisted reproductive technology (ART) [5]. In some other developed countries, this figure can reach 7% [3,6]. However, while conventional *in-vitro* fertilisation (IVF) provides effective treatment for many infertile couples, several conditions, including severe male infertility (accounting for 19–57% of cases), remain untreatable with a normal IVF cycle [7]. In such cases, intracytoplasmic sperm injection (ICSI), a more sophisticated technique in which an individual sperm is injected directly into the oocyte [8], is implemented and has been shown to be an effective approach for many cases. Despite this revolutionary approach, it is estimated that 1–5% of ICSI cycles still fail [6,9]. Considering that approximately 52% of all IVF cycles in the UK involve ICSI [5], this represents a significant problem, and on average would affect over 1000 couples per year in the UK alone. Indeed, despite the global expansion of ART, pregnancy and delivery rates following IVF and ICSI protocols rarely exceed 32% and 33%, respectively [10]. Consequently, there is constant need for a greater understanding of the molecular and physiological mechanisms underlying infertility, such as ICSI failure and other idiopathic conditions, such that the ART protocols can be improved, refined, or replaced. It is the intention of this review to discuss how investigative research into the process of oocyte activation has led to significant potential to both enhance existing ART protocols and provide the possibility of novel diagnostic tests for certain types of male factor infertility.

### Oocyte activation

IPrior to fertilisation, mature oocytes remain arrested in the metaphase stage of the second meiotic division (MII) [11]. Arrest is maintained by stabilisation of M-Phase promoting factor (MPF), the universal driver for G2/M cell cycle transition. MPF is a heterodimer consisting of a regulatory cyclin subunit, cyclin B, and a catalytic subunit, Cdc2 kinase. Active Cdc2 drives entry into M-phase by phosphorylating substrates leading to nuclear envelope breakdown and spindle formation [12]. Upon fusion with a sperm, MII arrest is alleviated, thereby allowing cell cycle progression, cell division, and embryogenesis to proceed. Release of meiotic arrest occurs via a series of concurrent events, collectively termed ‘*oocyte activation*’, which convert the oocyte into a totipotent zygote, able to form all types of body cells [3,13]. These morphological and biochemical events include cortical granule (CG) exocytosis to prevent polyspermy, extrusion of the second polar body, maternal RNA recruitment, male and female pro-nuclear (PN) formation, and the initiation of embryonic gene expression [14-20].

**Figure 1:**
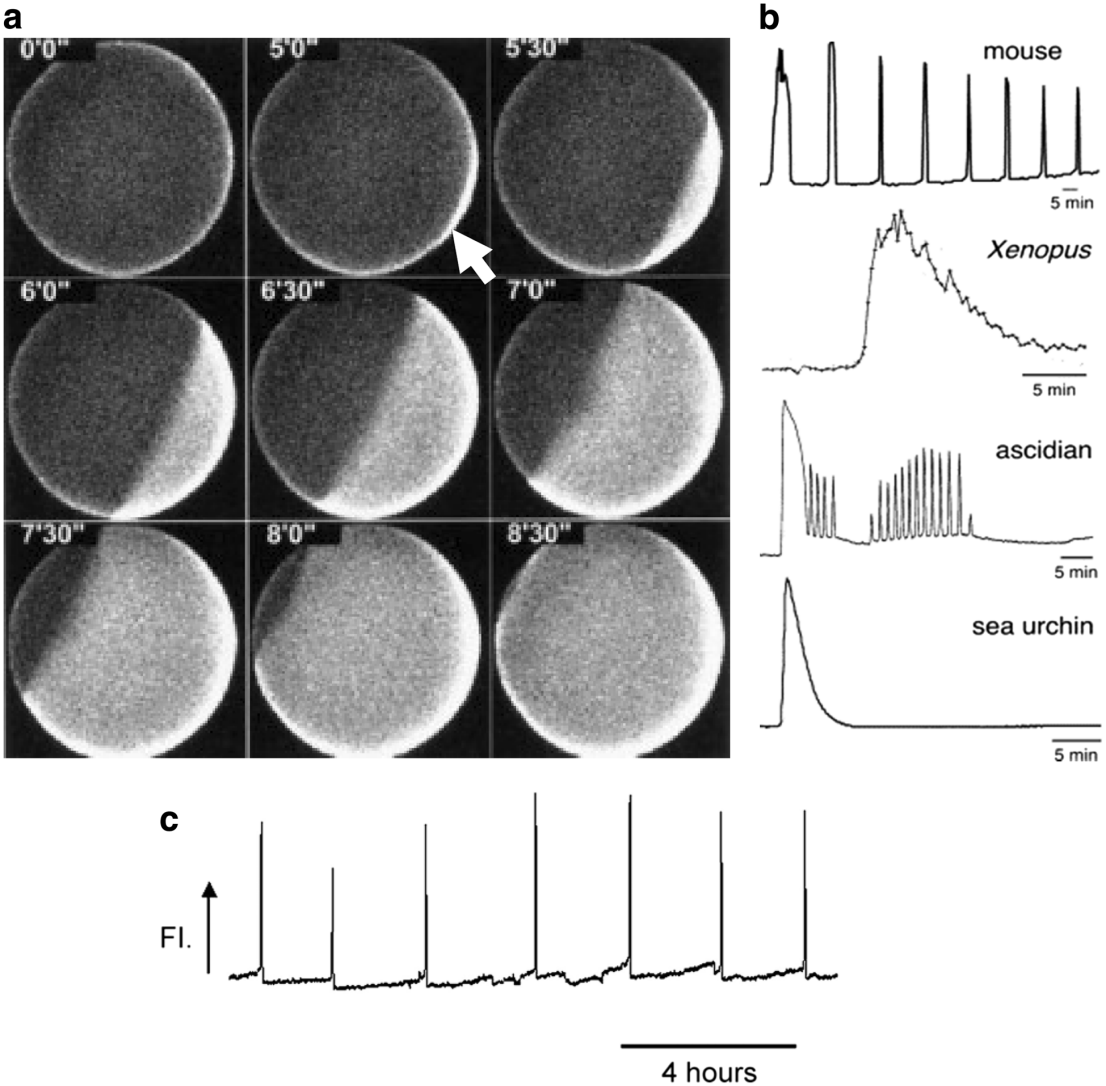
**A) Calcium wave as observed using calcium-green-1-dextran in a*Xenopus*egg.** Time 0 shows the egg’s resting levels of calcium. The sperm-induced calcium wave was initiated by sperm entry (indicated by white arrow), and traversed the entire egg. **B**) Ca^2+^ responses at fertilisation in eggs and oocytes of several species. **C**) Ca^2+^ release profile observed during human fertilisation. Reproduced from Fontanilla and Nuccitelli (1998) and Miyazaki (2006) with permission. Figure 1C reproduced from Rogers *et al*. (© Society for Reproduction and Fertility, 2004), with permission

Early research utilised calcium (Ca^2+^) sensitive dyes to demonstrate that non-mammalian oocytes, such as those from sea urchins and frogs, were activated by a single cytosolic Ca^2+^ transient initiating from the point of sperm entry and traversing across the egg to the opposite pole. In contrast, mammalian oocytes exhibit a series of Ca^2+^ ‘oscillations’ (Figure 1) [12,14,15,21-23]. These changes in cytosolic Ca^2+^ are now thought to be the universal trigger for oocyte activation and development [14,19,24]. Indeed, microinjection of Ca^2+^ ions alone triggered embryo development to the blastocyst stage in mice [23,25]. However, the nature, amplitude, duration, and frequency of this vital signal are species- specific [15-17,26].

In mammalian oocytes, Ca^2+^ oscillations are now generally accepted to be the direct result of inositol trisphosphate (IP_3_)-mediated Ca^2+^ release from intracellular Ca^2+^ stores Isuch as the endoplasmic reticulum (ER) [23,27-38]. Blocking, down-regulating, or reducing the expression levels of IP_3_ receptors (IP_3_Rs) in mouse and hamster oocytes, all inhibit Ca^2+^ oscillations and thus oocyte activation [39-42]. Furthermore, cytosolic increases in IP_3_ concentration during fertilisation in mammalian oocytes [23] provided further support for the importance of IP_3_ levels and IP_3_-mediated Ca^2+^ release.

Oocytes are remarkably sensitive to the specific frequency and amplitude of the Ca^2+^ oscillations evoked. The initial increase in intracellular Ca^2+^ appears to be critical for the initiation of both early and late events of oocyte activation [14,17,43], as well as exerting peri-implantation effects upon gene expression and development to term [17,44-46]. Many early activation events of physiological importance are not simultaneously initiated following the initial Ca^2+^ transient, but occur in a temporal order [17,45] through spatial and temporal regulation of periodic Ca^2+^ release [47]. The frequency and amplitude of Ca^2+^ oscillations influence cell cycle progression rates and protein expression profiles in early mouse embryos [16], and embryonic development in rabbits [19,48]. Considering that the rate of progression to the 2- and 4-cell stages of human oocytes following fertilisation has been proposed as an indicator of normal embryogenesis [49], the profile of Ca^2+^ oscillations at fertilisation may not only be necessary for oocyte activation, but may also be equally important for subsequent embryogenesis. Indeed, alterations in the Ca^2+^ oscillatory activity of vitrified mouse oocytes were found to directly affect oocyte quality and subsequent embryonic development [50].

Many theories have attempted to explain how the fertilising sperm elicits Ca^2+^ oscillations following mammalian gamete fusion [24,32,33,35,51]. One such model relates the interaction of the sperm with a coupled receptor on the plasma membrane of the oocyte, which in turn stimulates the release of Ca^2+^ either through a G-protein linked cascade or via tyrosine kinase activity. Indeed, this ‘membrane/receptor’ model was the dominant theory for many years [35,52]. However, despite many targeted studies, a sperm ligand/oocyte receptor of this nature still remains to be characterised [35]. Critically, the successful nature of microinjection techniques such as ICSI casts significant doubt over the membrane receptor theory as this technique bypasses sperm/oocyte membrane interaction completely.

A second model proposed that a soluble oocyte activation factor is released into the oocyte following gamete fusion, and subsequently interacts with ooplasmic components, ultimately leading to Ca^2+^ release. Indeed, the injection of sperm extracts into the oocytes of a variety of species, including marine worms, and ascidians, resulted in successful Ca^2+^ release and oocyte activation [15,53,54]. Sperm extracts from frogs, chickens, and tilapia fish, also trigger Ca^2+^ oscillations in mouse oocytes [55-57], suggesting the existence of a similar sperm-based mechanism throughout a wide spectra of species. A considerable body of research suggested that the IP_3_-dependant nature of oocyte activation depended upon a phospholipase C (PLC)-mediated mechanism [35], suggesting that the oocyte activation factor was a sperm-based PLC [26,48,58]. This supported the notion that the soluble sperm factor was a PLC which mediated the hydrolysis of phosphotidylinositol 4,5-bisphosphate (PIP_2_) to IP_3_ and diacylglycerol (DAG), leading to the activation of signalling pathways mediated by protein kinases, such as protein kinase C (PKC) [23,39,59]. General consensus agreed that the soluble factor responsible for Ca^2+^ release within oocytes was sperm-specific, as extracts from other tissues did not result in Ca^2+^ induction upon oocyte injection [53,60]. However, the specific identity of such a PLC isozyme remained a mystery for some time.

### PLCζ as the putative oocyte activation factor

Using mouse express sequence tag (EST) databases, Saunders *et al*. (2002) successfully identified a novel testis-specific PLC, termed PLCzeta (PLCζ), a ~74 kDa protein which was proven to play a key role in oocyte activation [59]. Subsequent studies have identified further mammalian PLCζ orthologues in human, hamster, monkey, and horse sperm [59,61-65], as well as in non-mammals such as chicken, fish, and quail [57,66-68].

**Figure 2:**
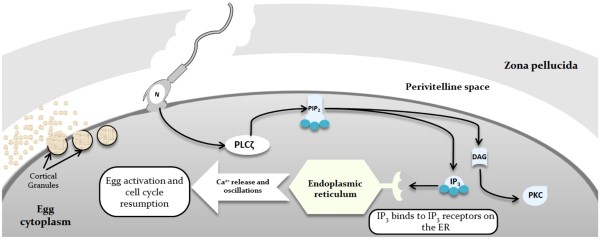
**Schematic representation of the current understanding of PLCζ mechanism of action.** Following sperm/oocyte fusion, PLCζ is released into the cytoplasm of the oocyte, where it facilitates the hydrolysis of membrane-bound PIP_2_ to DAG and IP_3_, triggering Ca^2+^ release from intracellular Ca^2+^ stores, leading to Ca^2+^ induced Ca^2+^ release and oocyte activation. The schematic illustrates the previous theory of PLCζ action upon membrane-bound PIP_2_, which is now being considered to be organelle-bound. N: Nucleus; PIP_2_: Phosphotidylinositol 4,5-bisphosphate; IP_3_: inositol trisphosphate; DAG: diacylglycerol; PKC: Protein kinase C; ER: Endoplasmic reticulum

Numerous studies support the contention that PLCζ is the physiological agent responsible for IP_3_-mediated Ca^2+^ release in activating oocytes (Figure 2) [18,59,61]. Micro-injection of both PLCζ cRNA and recombinant protein into mouse and bovine oocytes resulted in the initiation of oscillations and oocyte activation, similar to that seen during normal fertilisation, and stimulated embryonic development to the blastocyst stage [59,61,69-74]. Injection of human PLCζ cRNA also initiated Ca^2+^ oscillations in human oocytes and led to pre-implantation development [75]. Depletion of PLCζ from sperm extracts [59], or the inhibition of PLCζ expression in sperm using transgenic RNA interference [76], significantly abolished the ability to initiate Ca^2+^ release, or caused premature termination of Ca^2+^ oscillations and reduction in litter size.

Interestingly however, Coward *et al*. (2011) recently identified ovarian and brain isoforms of PLCζ in puffer-fish (*Takifugu rubripes*) [68]. Injection of cRNA corresponding to the ovarian isoform into mouse oocytes did not induce Ca^2+^ oscillations [68]. It is possible that puffer-fish PLCζ requires further oocyte- or sperm-based factors for Ca^2+^ inducing activity. Intriguingly, expression of puffer-fish PLCζ was also observed within the brain. This was particularly surprising considering that PLCζ is generally reported to be testis-specific [68]. While, Yoshida *et al*. (2007) reported the expression of PLCζ mRNA in the brain of both male and female mice [77], this directly contradicted the findings of Saunders *et al*. (2002) [59]. While it would be of interest to investigate the physiological relevance of PLCζ isoforms in the brain, it would first be necessary to confirm these earlier findings using more rigorous methodology [68].

Although it is possible that the ovarian PLCζ isoform in puffer-fish is an evolutionary adaption related to reproductive strategy, this discovery was particularly interesting given the debate regarding the mechanism responsible for oocyte activation [19,35,68,78]. Recent studies have proposed alternative sperm factor candidates apart from PLCζ, which are able to induce meiotic progression or the typical pattern of Ca^2+^ oscillations in a variety of species. Sette *et al*. (1997) previously identified the truncated c-kit (tr-kit), enriched in mouse sperm, as being an oocyte activation factor demonstrating meiotic resumption and mouse oocyte activation on microinjection of extracts expressing recombinant tr-kit [79]. Harada *et al*. (2007) identified citrate synthase as the major component responsible for egg activation in the newt *Cynops pyrrhogaster*[80], however, citrate synthase is not a proposed oocyte activation factor in mammals. Wu *et al*. (2007) also reported another possible candidate for the sperm factor, residing in the post-acrosomal sheath region of the perinuclear theca, termed post-acrosomal sheath WW domain-binding protein (PAWP), in bovine sperm and other mammalian species [81]. Despite PAWP and citrate synthase representing alternative candidates to PLCζ as the oocyte activation factor, the precise molecular mechanisms underlying the function of both proteins remain unknown, with no demonstration of the proteins triggering Ca^2+^ oscillations in mammalian oocytes casting doubt over their candidacy of being the mammalian oocyte activation factor [82]. While it is possible that oocyte activation involves the collective action of PLCζ and other sperm factors, such theories remain to be established [3]. Furthermore, it is also possible that oocyte-borne factors, similar to puffer-fish PLCζ, may contribute towards the activation mechanism.

**Figure 3:**
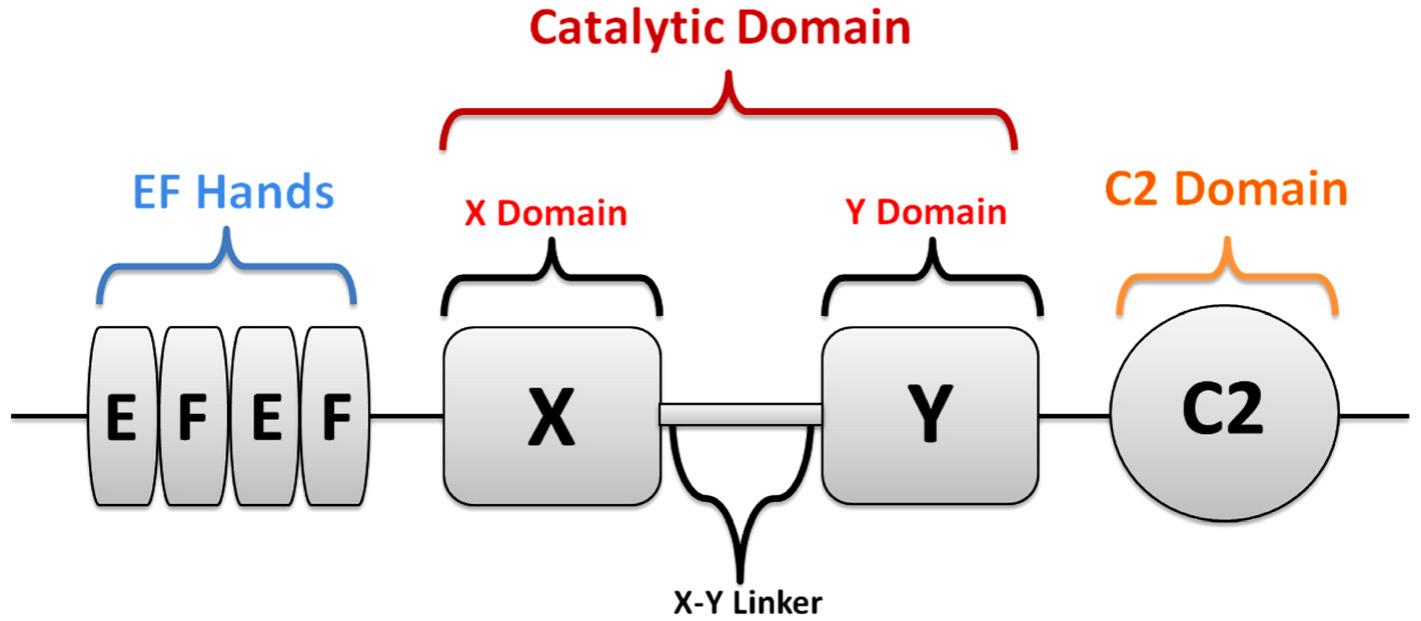
Schematic representation of PLCζ domain distribution.

Current data provides evidence that PLCζ represents a factor that is crucial to the process of mammalian oocyte activation. Consequently, this protein has been the target of a series of molecular and biochemical studies over recent years. PLCζ exhibits a typical PLC domain structure [59] with characteristic X and Y catalytic domains which form the active site in all PLC isoforms (β, γ, δ, ϵ and η) [83-85], a single C2 domain and four tandem EF hand domains. PLCζ exhibits closest homology with PLCδ, with 33% similarity [59]. However, one major difference to other PLCs is the absence of pleckstrin homology (PH) and Src homology (SH) domains, making PLCζ the smallest known mammalian PLC with a molecular mass of ~70 kDa in humans and ~74 kDa in mice (Figure 3) [59]. Another distinctive feature of PLCζ is its high sensitivity to Ca^2+^[69]. The catalytic XY domain is highly conserved, showing close homology with PLCδ, and mutagenesis of active site residues leads to total loss of Ca^2+^-oscillatory ability [33,86-88]. However, the X-Y linker region is poorly conserved amongst PLCζ isoforms, except for the presence of positively charged amino acids, prompting speculation that differing motifs in this region may underlie species-specific patterns of Ca^2+^ oscillations amongst mammals [33,36,89]. Nomikos *et al*. (2011) recently suggested that these charged amino acids may play an important role in the interaction of PLCζ with PIP_2_[87,90,91]. Yu *et al*. (2011) further demonstrated that whilst PLCδ targets PIP_2_ at the oolemma, mouse PLCζ appeared to target intracellular membranous PIP_2_ on distinct vesicular structures within the mouse oocyte cortex [92]. Moreover, fluorescently-tagged human recombinant PLCζ indicated localisation to discrete regions within the cell cytoplasm when expressed in HEK293T cells, adjacent to the nuclear envelope, possibly representing a specific organelle sub-type [74]. In accordance with these findings, mouse PLCζ remains in an inactive state and fails to induce Ca^2+^ oscillations in the CHO cell line, which are known to be devoid of organelle-bound PIP_2_[93].

As well as providing evidence for the Ca^2+^ releasing target of PLCζ, this also suggests that specific factors within the ooplasm may be required for PLCζ-mediated Ca^2+^ release [93]. However, Kashir *et al*. (2011a) demonstrated that recombinant human PLCζ was expressed in an active state within HEK293T cells, and maintained activity following injection of transfected HEK293T lysates into mouse oocytes [74]. Furthermore, there was a clear delay between lysate injection and the initiation of Ca^2+^ oscillations, possibly indicating that oocyte-based mechanisms resulted in the modification of recombinant PLCζ, ultimately resulting in an active state [74]. It is possible that these differences are due to inter-species variance, or the type of mammalian cells deployed in laboratory-based experiments. Indeed, human PLCζ has been shown to be much more potent in terms of PLC activity compared to the mouse isoform [3,33,35,59,61,71]. It also remains possible that HEK293T cells contain molecular mechanisms not present within CHO cells, which ultimately led to the presence of active PLCζ. Collectively, these studies provide significant insight into the mechanism of action of PLCζ, and demonstrate the importance of continued research effort, particularly with regard to the activation of human oocytes.

A sequence of amino acids predicting a nuclear localisation signal (NLS) was identified in the X-Y linker region of mouse PLCζ, thought to be a binding site of the nuclear transport receptor, which may play a role in regulating PLC activity. The distribution of PLCζ within the activating mouse oocyte changes during the first cell cycle, exhibiting PN localisation during zygotic interphase [66,94-97] thereby restricting access of the enzyme to its substrate PIP_2_, and thus resulting in termination of Ca^2+^ oscillations. Mutation of this putative NLS sequence prevented nuclear accumulation, as well as the termination of oscillations at interphase [33,95]. Kuroda *et al*. (2006) hypothesised that PLCζ is driven into the nucleus primarily by the NLS [98]. Close contact with EF/C2 domains appears to be required not only for enzymatic activity but also for nuclear translocation ability [69,96,99,100].

However, the injection of human, bovine, rat or medaka fish PLCζ into mouse oocytes does not result in PN translocation [64,66,97]. It is not yet clear whether human PLCζ has a distinctive NLS sequence, although nuclear localisation may prove to be the result of a conformational change or modification arising from interaction with other, as yet unknown, adaptors. It is worth noting that Ito *et al*. (2008) demonstrated nuclear localisation of human and mouse PLCζ in COS-7 cells, and that Coward *et al*. (2006) observed localisation of mouse PLCζ to either the nucleus or cytoplasm of COS cells [101]. In a more recent study, Kashir *et al*. (2011a) detected cytoplasmic localisation of human PLCζ when expressed in HEK293T cells [74]. While there seems to be conflicting evidence in this regard, the observed lack of nuclear localisation could reflect either the cell type used, or the reduced nuclear localisation activity of human PLCζ compared to mouse. It may also be the case that functional disparities may exist between the mouse NLS, as well as any potential human NLS. However, the existence, and functional significance if any, of the human PLCζ NLS must be confirmed before any such comparison can be made.

Upon PN envelope breakdown during entry into mitosis, Ca^2+^ oscillations resume [11,94-96,102] and may act in conjunction with modifications to the IP_3_ receptor to terminate Ca^2+^ signals at fertilisation [31,95]. Sone *et al*. (2005) showed that this pattern of PLCζ expression continues beyond first mitosis by exogenously expressing PLCζ at later stages of embryo development, showing continued nuclear sequestration of PLCζ during mitotic proliferation [103]. Thus, shuttling of PLCζ between the cytoplasm and nucleus appears to be strongly related to the termination/resumption of Ca^2+^ oscillations in a cell-cycle dependent manner, suggesting a possible role for PLCζ in mediating the cell-cycle dependency of Ca^2+^ oscillations in the early embryo [103]. Since no other species share the ability of mouse PLCζs for nuclear translocation, it remains unclear whether this is a widespread phenomenon in mammals, or whether it holds any physiological importance.

PLCζ is thought to remain in an inert state within sperm, and once introduced into the oocyte by gamete membrane fusion, becomes enzymatically active [33]. It is currently unclear how such a physiologically active protein is maintained in an inert form within sperm. Studies indicate that there may be an oocyte component that is necessary for the activation process [74,77,93]. Elucidation of what the oocyte factors are may explain how PLCζ is maintained in an inactive state in the sperm but is activated on release into the oocyte. It is possible that PLCζ needs to undergo post-translational modifications before reaching an active state [64]. Indeed, spontaneous proteolysis of PLCζ was apparent in both mouse and porcine sperm [89,104], with cleaved products re-associating to form functional heterodimers with increased levels of activity [89]. Moreover, a 62 kDa PLCζ isoform lacking EF1-3 hand domains was previously described in mouse sperm [69,89,99,104,105], and reported to activate mouse oocytes and support normal development to term [100]. Intriguingly, purified recombinant mouse PLCζ did not undergo cleavage, retained activity *in-vitro*, but was far less efficient at inducing Ca^2+^ oscillations in mouse oocytes [69,99,105]. It is vital, therefore, that future studies elucidate whether post-translational or covalent modifications such as cleavage are a necessary requirement for endogenous PLCζ activity.

There appears to be significant species-specific differences in the concentration and activity of PLCζ delivered into oocytes during fertilisation, allowing for the different specific requirements of each species, and the specific Ca^2+^ oscillation pattern leading to embryo development [64]. PLCζ has been detected in sperm from many species, and is localised to distinct regions in the sperm head, with suggestions of differential functional roles for each population [3,63,106]. Three distinct populations of PLCζ have been identified in the human sperm head; acrosomal, equatorial and post-acrosomal [85,106,107], whereas in mouse and boar sperm, two populations have been identified – acrosomal and post-acrosomal [63,108,109]. In equine sperm, PLCζ was reported to be localised to the acrosome, equatorial segment and head mid-piece, as well as principle piece of the flagellum [65]. In the pig, PLCζ was identified in the post-acrosomal region, and the tail [110].

**Figure 4:**
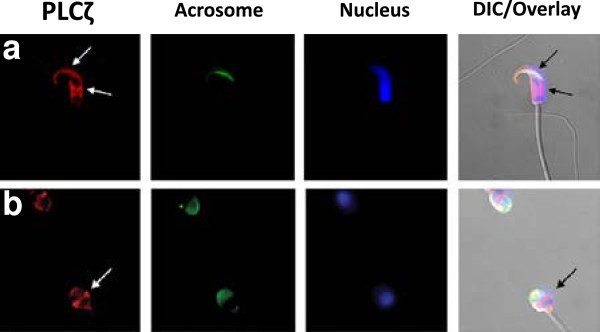
**Immunofluorescent localisation of PLCζ (red) in (A) hamster and (B) human sperm.** Fluorescein isothiocyanate-conjugated peanut agglutinin (FITC-PNA)-lectin staining (green) identifies the acrosome, and Hoescht-33342 staining (blue) identifies the nuclei. Arrows indicate the immunolocalisation of PLCζ within the acrosome and equatorial segment of hamster and equatorial segment of human sperm. DIC: differential interference contrast. Reproduced from Young *et al*. (2008) and Grasa *et al*. (2008) with permission

Young *et al*. (2009) demonstrated dynamic changes of PLCζ localisation in mouse sperm following capacitation and the acrosome reaction [63]. Prior to capacitation, both populations were present, with the acrosomal population being more prominent, and following capacitation, sperm showed post-acrosomal localisation only. Grasa *et al*. (2008) echoed the findings of Young *et al*. (2009) in capacitated and non-capacitated fertile human sperm, but described three distinct populations of PLCζ suggesting differential roles for each population [106] (Figure 4). Furthermore, Bi *et al*. (2009) identified an isoform of PLCζ, which they inexplicably termed NYD-SP27, found in the acrosome of human and mouse sperm [111]. These authors suggested that this protein was necessary for capacitation and the acrosome reaction, functioning as an intrinsic decapacitation factor. However, further evidence of this intriguing possibility has yet to be forthcoming.

Grasa *et al*. (2008) indicated that PLCζ was predominantly localised to the equatorial region of human sperm, with relatively smaller populations in the acrosomal and post-acrosomal regions [106]. While it is not yet clear whether a particular pattern of localisation is required, or whether a combination of different populations is required for functional ability, the equatorial and post-acrosomal populations would indeed permit rapid access to the ooplasm following sperm-oocyte fusion [112-114]. Indeed, post-acrosomal populations of PLCζ have also been identified in mouse, hamster, bull, pig, and boar sperm [63,102,106,108-110]. Furthermore, the presence of the post-acrosomal population of PLCζ following *in-vivo* induced acrosome reaction provides further credence to a specific role for this protein in oocyte activation, as the factor responsible would be expected to be retained following this important physiological process [63,106].

It has yet to be confirmed as to whether the population of PLCζ in the acrosomal region of sperm plays a role in the acrosome reaction as some studies suggest [63,106,111]. Aghajanpour *et al*. (2011) suggest that the localisation of PLCζ to the acrosome may be independent of PLCζ mRNA expression, suggesting that the observed reduction in expression of PLCζ mRNA in globozoospermic sperm may be related to other factors and not absence of the acrosome [115]. While it is possible that the acrosomal population plays a role in fertilisation, questions regarding the timing of the acrosome reaction relative to sperm-oocyte fusion [116] indicate that further work is required before such assertions are made. Understanding the relative roles of differential populations of PLCζ will be essential prior to the use of this protein clinically, and should thus be the focus of future research directives. However, it is important to note that the polyclonal nature of all PLCζ antibodies used thus far may explain the variance in localisation patterns observed between existing studies. Indeed, Aarabi *et al*. (2012) recently suggested PLCζ localisation to only the acrosomal and post-acrosomal regions of mouse, boar, and human sperm [117]. While the specificity of this particular antibody was previously confirmed by Grasa *et al*. (2008) in human sperm [106], immunoblotting results from the study by Aarabi *et al*. (2012) also appeared to identify cross-reactivity with unidentified proteins other than PLCζ [117]. Furthermore, considering that Grasa *et al*. (2008) suggested variance in predominant localisation patterns amongst fertile males (sperm from 6 human males), specific conclusions cannot be drawn from the relatively smaller sample size (sperm from 3 human males and 1 mouse) examined by Aarabi *et al*. (2012). However, it is imperative that efforts are directed towards the synthesis of more specific monoclonal antibodies against PLCζ before conclusive remarks regarding variant isoforms and localisation patterns of PLCζ may be made.

PLCζ mRNA expression and the initiation of translation have been identified during early and late stages of spermatogenesis in mice and pigs [62,63,65,109,118]. PLCζ mRNA has also been identified in ejaculated human sperm [115,119-121]. While injection of PLCζ cRNA into the oocyte induces the production of PLCζ protein [59], the functional role of PLCζ mRNA in the zygote remains to be ascertained. Considering there is a considerable difference in sperm mRNA content between fertile and infertile individuals [122], it seems plausible to consider that similar differences may apply to PLCζ, with consequential repercussions on fertility.

### Links between PLCζ and male-factor infertility

Deficiency in the mechanism of oocyte activation is regarded as the principal cause of fertilisation failure following ICSI, accounting for an estimated 40% of failed cases [3,123-125]. However, ICSI addresses only one of the necessary requisites for successful fertilisation, that of sperm penetration, whereas a range of post-penetration events are crucial for successful fertilisation [126]. The aetiology of ICSI failure in such cases is likely to be multi-factorial in nature, and may be attributable to factors in the oocyte since the inherent quality of the oocyte is of great importance for successful fertilisation. Such factors would thus be reliant upon the fidelity of oocyte maturation, such as the inadequate sensitisation of IP_3_ receptors and ER Ca^2+^ concentration regulation required for optimal sperm-initiated Ca^2+^ release [11,127-129], or a reduction in the number of CG due to premature release, resulting in early blockade to polyspermy prior to insemination [20,130]. The suggestion that oocyte quality plays a role in fertility is further strengthened by a recent study reporting that the process of vitrification in ART affects the distribution of IP_3_Rs, with subsequent detrimental effects upon Ca^2+^ oscillatory activity and embryo development, thus providing an explanation for low rates of blastocyst formation following ICSI treatment using vitrified oocytes compared to fresh oocytes [50].

**Figure 5:**
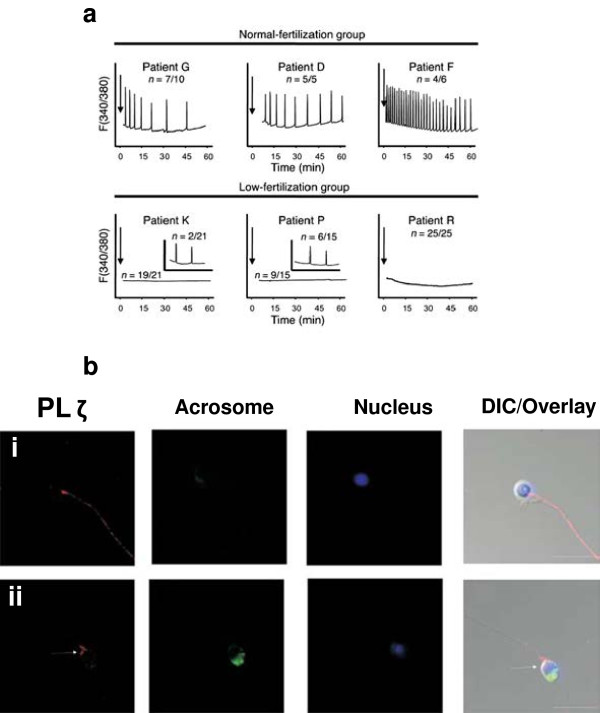
**A) Ca**^**2+**^** oscillation profiles following the injection of sperm from normal fertile humans (top panel) and infertile patients who had previously failed or exhibited low fertilisation rates following ICSI (bottom panel).** Arrows denote time of sperm injection, and *n* indicates the number of mouse oocytes exhibiting the corresponding Ca^2+^ oscillation profile. B) Reduced PLCζ immunostaining (red) in sperm from infertile ICSI failed patients exhibiting abnormal morphology (in this case globozoospermia; acrosome-less, round-headed sperm) (i), and normal morphology (ii). Fluorescein isothiocyanate-conjugated peanut agglutinin (FITC-PNA)-lectin staining (green) identifies the acrosome, and Hoescht-33342 staining (blue) identifies the nuclei. Arrow indicates reduced PLCζ immunolocalisation within the equatorial segment. Scale bars indicate 5 μm. Reproduced from Yoon *et al*. (2008) and Heytens *et al*. (2009) with permission.

Sperm defects however, are considered the leading cause of activation failure [3], and given the role of PLCζ as the oocyte activating factor; it is highly plausible that defective forms, or abnormal function, of PLCζ may be the underlying cause of certain types of male-factor infertility and oocyte activation failure. Indeed, evidence indicates that the severity of sperm defects, opposed to sperm source, determines the efficacy of ICSI success [131]. Much evidence now exists to highlight PLCζ’s role in fertility and relative role in male-factor infertility. Sperm from infertile men which consistently fail IVF and ICSI, also fail to induce Ca^2+^ oscillations upon injection into mouse oocytes, or cause abnormal patterns of Ca^2+^ release when compared with those of fertile males [86,107] (Figure 5A). Such sperm also exhibited absent/reduced levels, or abnormal isoforms, of PLCζ (Figure 5B) [86,107].

The first genetic link between PLCζ and oocyte activation deficiency (OAD) was reported by Heytens *et al*. (2009), who identified a substitution mutation in an infertile male diagnosed with OAD [86]. This involved a substitution of a histidine to proline within the Y domain of the catalytic site of the protein, at position 398 of the PLCζ open reading frame (PLCζ^H398P^). Micro-injection of PLCζ^H398P^ cRNA into mouse oocytes failed to induce Ca^2+^ oscillations, whilst the injection of sperm possessing this mutation resulted in an atypical pattern of Ca^2+^ oscillations [86]. An equivalent mutation was subsequently created for mouse PLCζ (PLCζ^H435P^) [88]; murine experiments confirmed that this mutation caused major structural changes to the PLCζ protein, resulting in functional inactivation.

**Figure 6:**
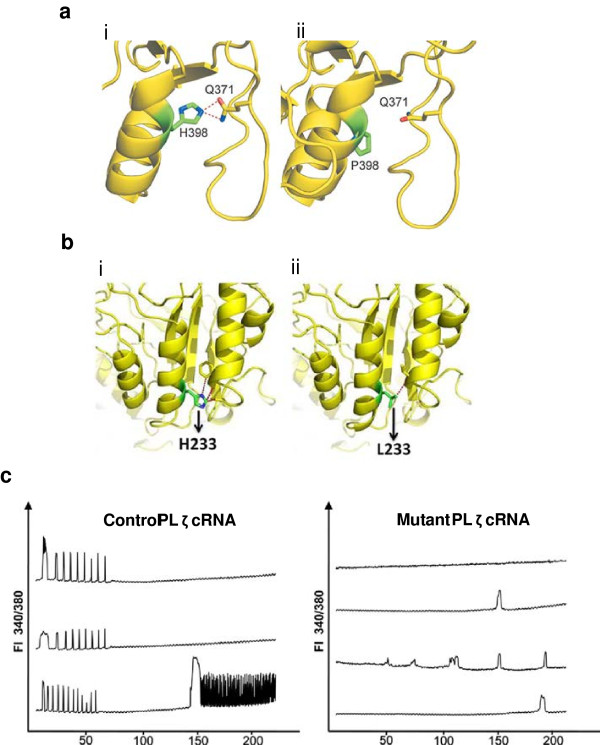
**Schematic representations of the effects of the Histidine > Proline (PLCζ**^**H398P**^**) (A) and Histidine > Leucine (PLCζ**^**H233L**^**) (B) point mutations identified by Heytens*et al*. (2009) and Kashir*et al*. (2012) in an infertile male patient diagnosed with oocyte activation deficiency.** For both A) and B), schematics represent local protein folding before mutation **(i)** and after mutation **(ii)**. **C)** Microinjection of wild type and mutant PLCζ into mouse oocytes and resulting calcium release patterns. FI 340/380: ratio of fluorescence at wavelengths of 340 nm and 380 nm. Reproduced from Heytens *et al*. (2009) and Kashir *et al*. (2012) with permission.

More recently, Kashir *et al*. (2012) identified a further novel point mutation from the same patient from whom the H398P mutation was first identified, involving a substitution of a histidine to leucine residue in the catalytic X domain at position 233 of the PLCζ open reading frame (PLCζ^H233L^) [132]. Micro-injection of PLCζ^H233L^ cRNA resulted in an abnormal Ca^2+^ release profile and like the H398P mutation, a failure to activate oocytes (Figure 6) [132]. Interestingly, this study also showed that both the PLCζ^H398P^ and PLCζ^H233L^ mutations, which are heterozygous in nature, originated from different parental origins: PLCζ^H398P^ was paternal in origin, while PLCζ^H233L^ was maternal. These findings represented the first description of an autosomal point mutation resulting in male fertility via the maternal lineage [132]. Furthermore, albeit speculation at present, Kashir *et al*. (2012) hypothesised that mutations in PLCζ may be recessive in nature, requiring mutation on both parental alleles for full infertility to occur. One would also reason that heterozygous mutations in PLCζ may result in cases of sub-fertility. Indeed, Kashir *et al*. (2011a) reported that HEK293T cells over-expressing fluorescently-tagged PLCζ^H398P^ exhibited a lower level of fluorescence compared to HEK293T cells over-expressing fluorescent-PLCζ^WT^, correlating to absent/severely reduced levels of PLCζ in sperm from the patient from whom the H233L and H398P mutations were identified [74].

Furthermore, absent/reduced levels of PLCζ in sperm have been implicated in forms of male infertility where such sperm is repeatedly unable to activate oocytes [86,107,133]. It is therefore possible that cases in which PLCζ is absent or severely reduced may be due to destabilising effects caused by mutation in highly conserved regions of PLCζ. However, studies examining the role of deletions in critical domains essential to mouse PLCζ function did not report any differences in protein stability [89,105]. Furthermore, considering that sperm and HEK293T cells may exhibit very different molecular mechanisms, it is important that future studies examine the effect of mutant PLCζ in testicular germ cells to investigate whether such a trend is repeated, and to determine whether such loss-of-activity mutations are potentially disruptive to the overall folding of PLCζ.

### The therapeutic potential of PLCζ for ART

Although ICSI results in average fertilisation rates of 70% [3,134], complete or virtually complete fertilisation failure still occurs in 1–5% of ICSI cycles [3,9,124,135], corresponding to approximately 1000 cases per year in the UK alone. The incorrect injection of sperm, expulsion of injected sperm from the oocyte, and the failure of sperm head decondensation are not considered to contribute substantially to fertilisation failure following ICSI [3,9]. A deficiency in the mechanism of oocyte activation is regarded as the principal cause of fertilisation failure, or abnormally low fertilisation after ICSI, and can recur in successive ART cycles [3,123,136]. At present, such cases can only be resolved using assisted oocyte activation (AOA).

**Figure 7:**
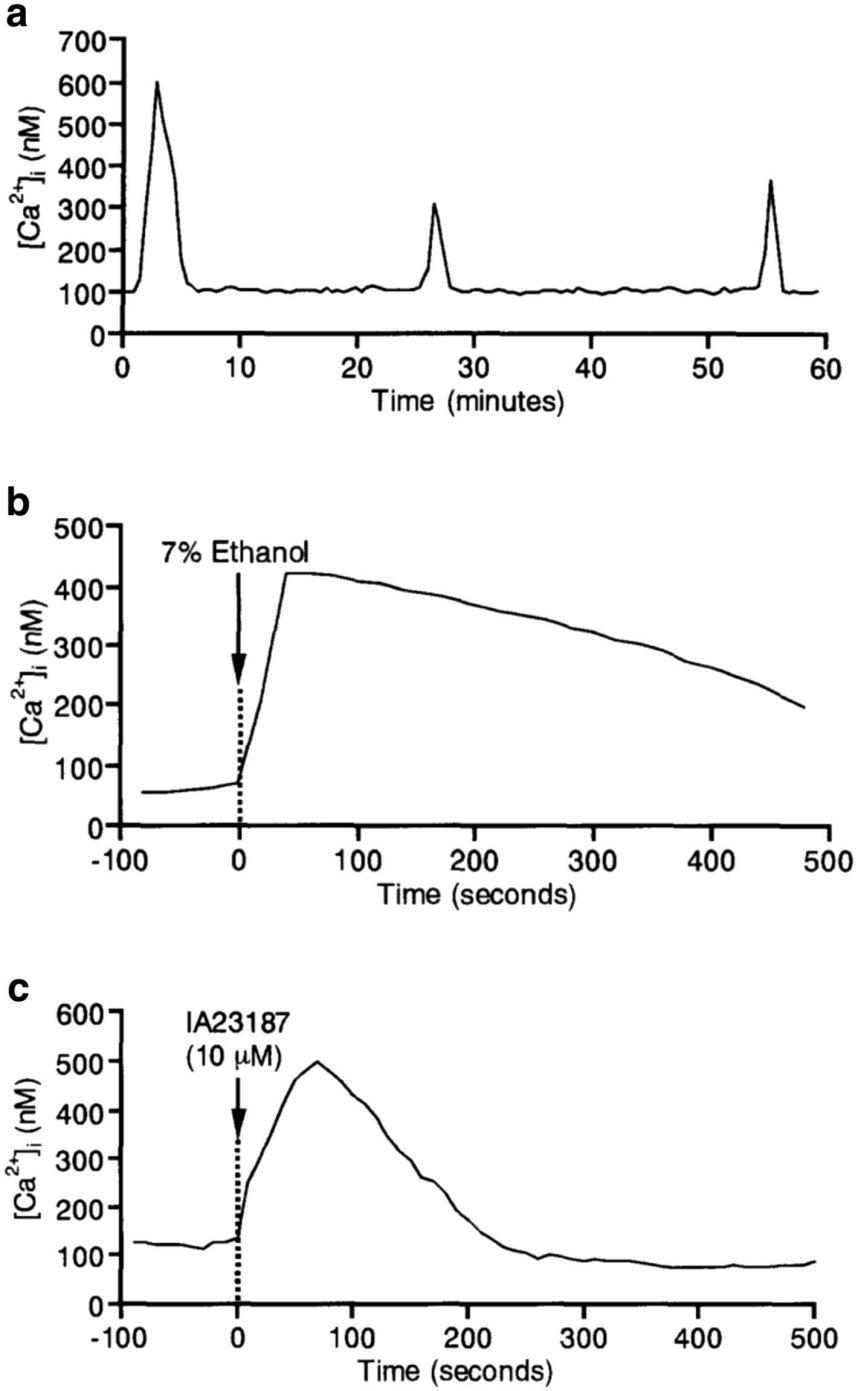
**Representative Ca**^**2+**^**responses of bovine oocytes treated with A) sperm (normal fertilisation), B) 7% ethanol, and C) Ca**^**2+**^**ionophore IA23187.** Reproduced from Nakada and Mizuno (1998) with permission.

Cases of oocyte activation failure can currently be treated by AOA methods such as Ca^2+^-ionophore or strontium chloride [9,133,136,137]. The most popular artificial activating agents for human oocytes include Ca^2+^ ionophores such as ethanol, ionomycin and A23187, electrical pulses, often in combination with protein synthesis or kinase inhibitors such as 6-dimethylaminopurine (6-DMAP) or puromycin that block the re-synthesis of cyclin B or CDK1 activity [136]. Taylor *et al*. (2010) demonstrated high rates of fertilisation, and successful pregnancy, in PLCζ-deficient globozoospermic patients using a Ca^2+^ ionophore to artificially activate the oocytes following ICSI [133]. However, there is significant concern as to how such chemicals may be detrimental to embryo viability and future health due to potential cytotoxic, mutagenic and teratogenic effects on oocytes and embryos [6]. Ca^2+^ release patterns following ionophore treatment do not mimic those observed during normal fertilisation (Figure 7) [86]. Consequently, the abnormal Ca^2+^ signal induced, which often manifests as a single Ca^2+^ transient, is a potential threat to ensuing development at later stages [45,138], with potential repercussions on epigenetic processes [139]. Moreover, the threat is increased in cases of AOA with abnormal sperm such as in cases of globozoospermia, due to the high degree of sperm DNA fragmentation associated with this pathology [133,139,140]. Other activating agents have been shown to induce multiple transients, such as strontium chloride in mice [14,141], phorbol esters [141] or thimerosal [142]. These, however, have only been reported to be efficient in a limited number of species, are less efficient than ionophores in most species, or can cause meiotic spindle disruption [143].

The most efficient of these is Strontium (Sr^2+^), which was found to elicit Ca^2+^ oscillations through the synergistic action of PLC activation and IP_3_ activity mediation via IP_3_ receptor sensitisation to release Ca^2+^[144], the use of which resulted in successful pregnancy of normozoospermic patients diagnosed with OAD [145]. However, Sr^2+^ treatment varies in terms of oocyte activation efficacy depending on the species in which it is used [9] with success rates being relatively low in humans [145]. Furthermore, little is currently known as to the downstream effects of Sr^2+^ treatment on signalling molecules such as PKC and DAG. It would, therefore, be prudent to investigate and develop new methods of AOA which elicit Ca^2+^ release in an endogenous manner, to circumvent these concerns for clinical scenarios. An ideal example would be a purified recombinant version of human PLCζ protein, the principle of which has been previously demonstrated by Yoon *et al*. (2008) using mouse cRNA [107].

**Figure 8:**
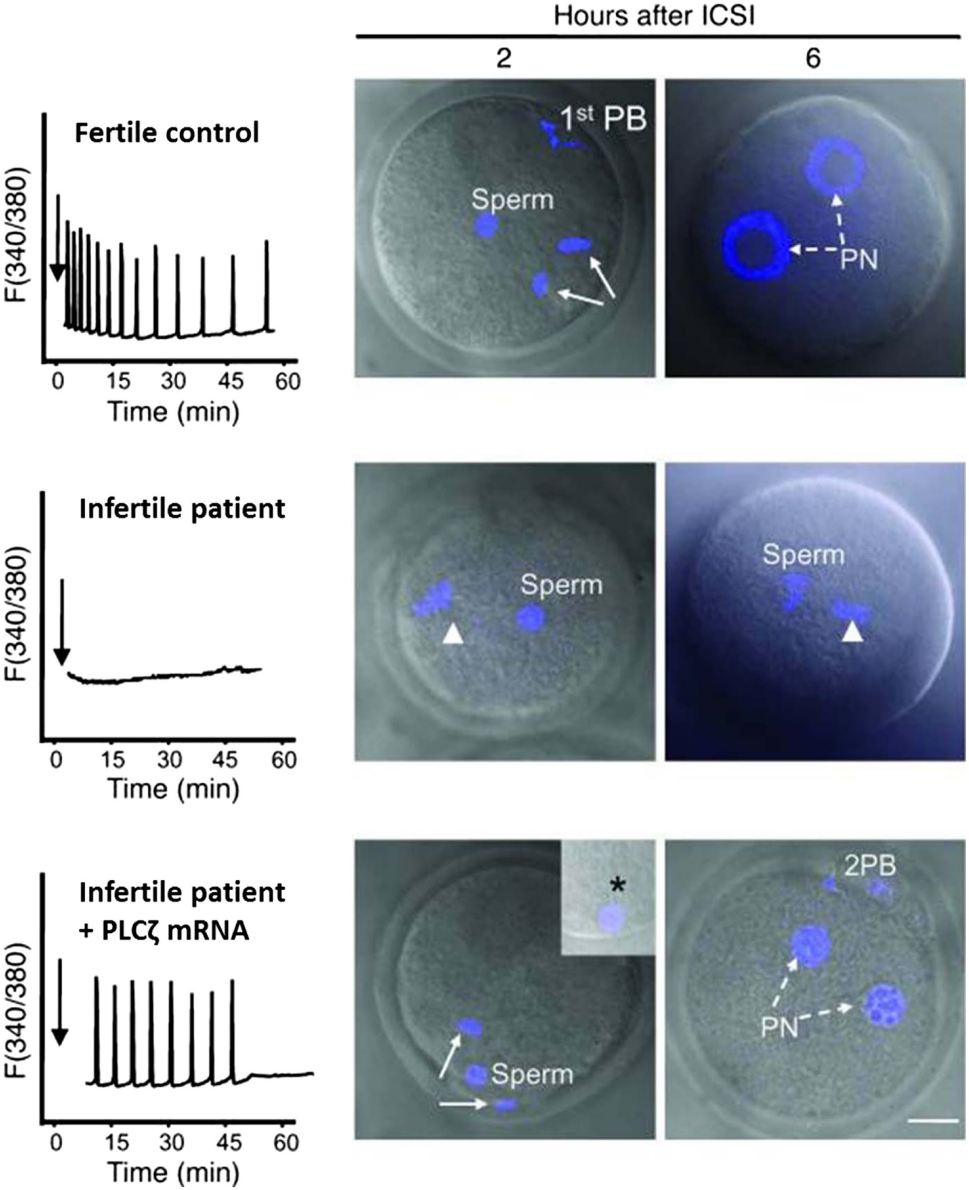
**Injection of a sperm from a fertile control elicits Ca**^**2+**^**oscillations and activates mouse oocytes (top panel).** Sperm from an infertile patient with a history of ICSI failure was unable to elicit Ca^2+^ oscillations, and was unable to activate mouse oocytes following injection (middle panel), but was able to do so following the co-injection of PLCζ mRNA (bottom panel). Arrows denote time of sperm injection. 1^st^ PB, first polar body; 2nd PB, second polar body. TO-PRO-3 staining (blue) stains chromatin. Asterisk in inset points to the persistence of the human sperm tail in mouse eggs. Scale bar indicates 10 μm. Reproduced from Yoon *et al*. (2008) with permission.

Yoon *et al*. (2008) showed that the failure of sperm exhibiting abnormal PLCζ localisation to activate an oocyte could be rescued upon co-injection with mouse PLCζ mRNA (Figure 8) [107], while Rogers *et al*. (2004) showed that it was possible to generate blastocysts parthenogenetically following the injection of PLCζ cRNA into human oocytes [75], providing significant support for the clinical use of PLCζ as a therapeutic. However, the therapeutic utilisation of PLCζ cRNA is not likely to be viable, since the uncontrollable transcription of PLCζ may be detrimental to normal pre-implantational development through gene expression irregularities, with developmental defects observed in some embryos [16,45,75]. Moreover, injected PLCζ RNA could potentially be reverse transcribed into cDNA which may then be incorporated into the genome [3,146]. It follows therefore, that an active, purified, recombinant version of PLCζ would be the ideal alternative for therapeutic application in the clinic, as it would function in a safe and endogenous manner.

Consequently, the synthesis of a pure and active recombinant form of PLCζ has been a key goal over recent years. However, this has proved far more difficult than first perceived. Purification, in particular, tends to be tenuous with previous studies culminating in an inactive protein following purification. This suggests either that the purification process itself renders the protein inactive, or that the laboratory processes involved did not permit key post-translational modifications or protein-folding mechanisms [64]. Kouchi *et al*. (2004) successfully purified a mouse recombinant PLCζ protein which exhibited high Ca^2+^ sensitivity and induced Ca^2+^ oscillations upon injection into mouse oocytes [69]. However, these oscillations were of a much higher frequency than those seen during normal fertilisation and have proved difficult to replicate thereafter. More recently, Phillips *et al*. (2011) demonstrated that a cell lysate prepared from CHO cells transfected with mouse PLCζ exhibited high recombinant protein expression, and on micro-injection into mouse oocytes, elicited Ca^2+^ oscillations representative of oocyte activation [93].

**Figure 9:**
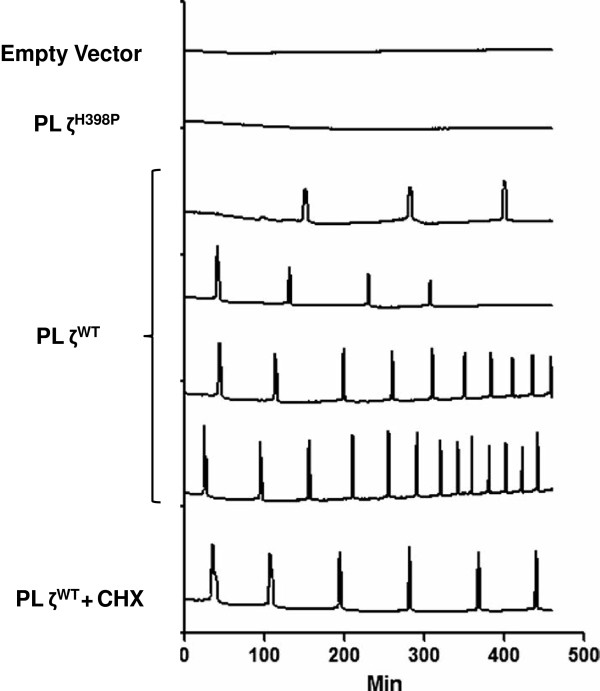
**Representative Ca**^**2+**^**oscillation profiles of mouse oocytes injected with lysates obtained from HEK293T cells transfected with PLCζ isoforms.** Baseline values for all Ca^2+^ traces are stacked to facilitate visualization of the responses. Empty vector: Ca^2+^ trace obtained from injection of lysates obtained from HEK293T cells transfected with vector without PLCζ; PLCζ^H398P^: Ca^2+^ trace obtained from injection of lysates obtained from HEK293T cells transfected with the H398P isoform of PLCζ; PLCζ^WT^: Ca^2+^ trace obtained from injection of lysates obtained from HEK293T cells transfected with wild type PLCζ; CHX: cyclohexamide (an inhibitor of RNA synthesis). Figure reproduced from Kashir *et al*. (2011a) with permission.

Until very recently, progress remained slow in extrapolating such techniques to the human protein. However, the use of a human cell line has finally yielded a cell lysate expressing a recombinant human PLCζ protein which, upon injection into mouse oocytes, elicited Ca^2+^ oscillations characteristic to those seen in normal fertilisation [74]. While representing a significant milestone in the clinical translation of PLCζ (Figure 9), there is still much to accomplish, given that activity was only evident in cell lysates, and not in a purified form.

Given that PLCζ plays a fundamental role in the process of oocyte activation, it is logical to deliberate how this knowledge might assist with the generation of a male contraceptive agent for fertile males, for example by specifically blocking the physiological action of PLCζ. However, this is not without complication and a significant body of research would be required to pursue this goal, including elucidation of the three-dimensional structure of the protein. Given that PLCζ has been detected in tissues and cells other than sperm in non-mammalian species [68], it would appear prudent to carry out wider scale expression studies to re-confirm that PLCζ is specifically expressed in human sperm and not any other cell or tissue-type. While inactivation of the PLCζ protein is theoretically possible, it is critical that new contraceptive agents are safe, reversible, effective, and specific in their mode of action upon target mechanisms. One particular caveat, is the fact that PLCζ RNA has been identified in ejaculated sperm [115,120,121], suggesting that even if PLCζ protein is inactivated in epididymal sperm, stocks of RNA remaining in the sperm may be able to contribute towards Ca^2+^ release [72,74,75,107]. While levels of PLCζ RNA present in sperm may not be sufficient to lead to complete oocyte activation, these levels may contribute towards the overall pattern of Ca^2+^ release. Indeed, injection of human PLCζ cRNA as low as 0.001 μg/μl and 0.02 μg/ml are able to result in Ca^2+^ release in mouse and human oocytes respectively [61,74].

### PLCζ as a prognostic biomarker of male fertility

Semen analysis based on concentration, motility and morphology has been used for the diagnosis of male fertility for many years, using criteria for normal semen parameters established by the WHO [147]. However, a significant number of patients with normal sperm parameters still undergo difficulty in achieving successful pregnancy [148]. This reflects the complexity of the events involved in sperm function, indicating that analysis based on concentration, motility and morphology may not represent an entirely accurate tool to assess the fertilising potential of a sperm sample. Consequently, there is significant interest at present for the development of additional tests to complement existing analytical procedures. Recent studies have demonstrated the potential of measuring sperm nuclear DNA fragmentation and protamine deficiency in sperm samples by revealing significant differences between fertile and infertile sperm [149,150].

It is conceivable that the quantitative analysis of PLCζ within sperm may represent a rapid and informative diagnostic tool for both research and clinical purposes, by providing an indicator, or biomarker, of oocyte activation ability. A heterologous ICSI model, the mouse oocyte activation test (MOAT), was previously developed to evaluate the activation capacity of human sperm by microinjection into mouse oocytes [3,134,136]. This assay represents a useful method with which to investigate cases of low ICSI success rates [106]. However, considering that human PLCζ is thought to be more potent in its activity compared to mouse PLCζ [33], and that oocytes from various species are thought to be ‘fine-tuned’ to specific levels and activity of PLCζ from that species [64], it is possible that the MOAT may only be useful to detect extreme cases of OAD where sperm is completely devoid of PLCζ, and not cases where only a more subtle reduction is evident. Of course, such tests require the provision of animal facilities and specialised skills which may not be routinely available for most ART centres, and may therefore be more useful in a research setting.

Identifying a pattern of localisation of PLCζ that is compatible with its role as the putative endogenous oocyte activation factor provides further confirmation of its physiological relevance as a mediator of this important process. Moreover, the identification of specific localisation patterns in fertile males, and their precise functional roles, would provide a key benchmark to which infertile sperm may be compared. Previous work using immunofluorescent techniques has already demonstrated that there appears to be a pattern of PLCζ localisation in the sperm head that is consistent with fertile sperm [86,106], and an abnormal pattern evident in ICSI-failed sperm [74,86,107], implicating a correlation between an abnormal localisation pattern of PLCζ with aberrant function/infertility. This suggests that by determining the expression, and localisation pattern, of PLCζ in sperm is likely to represent a credible index of oocyte activation ability for patients seeking ART.

Kashir *et al*. (2011b) investigated the effects of routine ART techniques upon the levels of PLCζ in fertile male donors, and revealed that cryopreservation, a common technique utilised for the preservation of fertility in patients undergoing fertility treatment as well as radio/chemotherapy or surgery [151], had a significant detrimental effect upon the levels of PLCζ compared with fresh sperm [152]. Furthermore, Nakai *et al*. (2011) showed that the pre-treatment of pig sperm reduced oocyte-activating ability via significant reductions in the levels of PLCζ levels compared with untreated sperm [110]. Interestingly, Nakai *et al.* (2011) also detected a population of PLCζ in the tail of pig sperm. Removal of the tail via pre-treatment methods reduced oocyte-activation ability, indicating that optimal ability was achieved via ICSI using only whole sperm [110]. Given that low PLCζ concentrations in sperm are linked with infertility, these studies further support the notion that PLCζ represents a beneficial biomarker for ART. Indeed, Kashir *et al*. (2011b) demonstrated that density gradient washing (DGW; a centrifugation method used to isolate the best quality sperm based on motility parameters) increased the proportion of sperm exhibiting PLCζ immunofluorescence in fertile male donors [152], thus increasing the likelihood of successful activation.

Since PLCζ mRNA has been identified in sperm [120,121], it is possible that the determination of PLCζ mRNA may represent another useful clinical indicator, although the precise function of this mRNA store is not yet known. Several studies have already demonstrated the differential expression of some key mRNAs in infertile males compared to fertile males [122,147,153], thus suggesting the potential for the development of a fertility index via relative expression analysis. Indeed, Kaewmala *et al*. (2011) indicated that higher levels of PLCζ mRNA in boar sperm correlated with better quality [109], while Aghajanpour *et al*. (2011) investigated whether the level of PLCζ mRNA in human sperm may be an indicator of the potential of a patient sample to induce oocyte activation [115]. The authors of the latter report identified a significant reduction in levels of PLCζ mRNA in individuals with low or failed fertilisation with ICSI, compared to fertile controls. It follows then that the relative expression of PLCζ mRNA may represent a credible biomarker of the oocyte activation ability of a given sperm sample. Indeed, several authors hypothesise that the future of male infertility diagnosis may rely heavily upon the use of microarrays to determine the expression levels of target mRNAs in discrete sperm samples [154-158].

Interestingly, a recent study revealed that by assessing the rhythmical cytoplasmic movements triggered by Ca^2+^ oscillations in the fertilised mouse oocyte using particle image velocimetry (PIV), it was possible to predict the developmental potential of the zygote [159], thus offering a potential viability index for oocytes fertilised *in-vitro*. Swann *et al*. (2012) further validated the concept of PIV analysis by demonstrating that the frequency of PLCζ-induced cytoplasmic Ca^2+^ oscillations and the pattern of cytoplasmic movements within aged ICSI-failed human oocytes were synchronous in nature [160]. Collectively, these studies indicate that the use of PIV to analyse cytoplasmic movements may represent an exciting non-invasive method for monitoring Ca^2+^ oscillation patterns in human oocytes in a clinical setting, thereby providing an early and effective indicator of zygote viability following IVF [159,160].

Increasing evidence supports the importance of oocyte factors in the fertilising potential of an oocyte. The oocyte undergoes cellular arrangement and modifications during maturation to optimise the internal environment for optimal Ca^2+^ release ability upon sperm fusion [128]. A dysfunctional environment within the oocyte, in the case of an aged oocyte for example, is very likely to impede the process of fertilisation [161]. Further understanding of the factors and mechanisms involved in creating a fully viable oocyte are equally as important as understanding the manner in which the fertilising sperm induces Ca^2+^ release upon gamete fusion. Important lessons can be gained via expression studies in the laboratory; for example, studies demonstrating successful Ca^2+^ release upon the expression of recombinant PLCζ in one specific cell line but not another cell type [74,93], indicate that there may be hitherto unknown oocyte components that are required for the activation process. Elucidation of the specific identities of such factors may explain how PLCζ is maintained in an inactive state in the sperm but activated upon release into the oocyte. Furthermore, evidence suggests that in addition to interaction with oocyte factors, PLCζ may undergo post-translational modifications before attaining the ability to induce Ca^2+^ release. Cooney *et al*. (2010) suggest that this is a key requirement for PLCζ to attain endogenous activity [64,74,162]. It is important that future studies aim to confirm whether post-translational modifications and/or proteolytic cleavage, are important for the functional role of PLCζ, and address how deficiencies in these processes may relate to an infertile state.

## Conclusions

It is clear that PLCζ plays a fundamental role in the activation of mammalian oocytes and that genetic, molecular or biochemical perturbation of this key protein is strongly linked to OAD and human infertility. Consequently, there is significant scope for our understanding of PLCζ to be translated to the ART clinic, both as a novel therapeutic agent with which to rescue OAD, or as a prognostic/diagnostic biomarker of oocyte activation ability in target sperm samples. However, there are several key target areas for future research that must be addressed. Firstly, although an active recombinant human PLCζ protein has been successfully synthesised in mammalian cell lysates [74], steps must now be taken to extend such progress to final purification, concentration, and activity testing. Such work underpins the successful deployment of recombinant PLCζ protein as a more physiological method of AOA compared to current protocols. Secondly, there is significant interest at present to identify novel biomarkers of sperm function to complement and extend current semen analysis procedures in the ART clinic. Initial findings indicate that PLCζ represents a useful prognostic/diagnostic biomarker of oocyte activation ability. However, several questions remain to be addressed before PLCζ may be competently utilised in this clinical capacity. For example, we cannot yet confirm beyond doubt whether a specific pattern of PLCζ localisation is required for successful oocyte activation, or whether the mere presence of PLCζ within a particular concentration window, regardless of localisation pattern, is all that is required. It is also necessary to address the potential effects that routine ART laboratory techniques may exert upon PLCζ concentration and function within human sperm, and whether such effects may inadvertently reduce fertilisation capacity in a clinical setting. It is imperative that future research adopts a range of experimental approaches in the research laboratory, but also allows for intensified screening within the clinical setting, which includes cases related to OAD, ICSI-failure, and idiopathic infertility. It is readily apparent that clinical data relating to PLCζ is still lacking, and that our current understanding is based on only a limited number of cases. However, given appropriate levels of research attention, it is already clear that PLCζ is likely to provide the ART clinic with significant improvements to prognostic and diagnostic testing, and safer options for therapeutic intervention.

## Abbreviations

WHO: World Health Organisation; ART: Assisted reproductive technology; HFEA: Human Fertilisation and Embryology Authority; IVF: In-vitro fertilisation; ICSI: Intracytoplasmic sperm injection; CG: Cortical granule; PN: Pro-nuclear; Ca2+: Calcium; IP3: Inositol trisphosphate; ER: Endoplasmic reticulum; PLC: Phospholipase C; PIP2: Phosphotidylinositol 4,5-bisphosphate; DAG: Diacylglycerol; PKC: Protein kinase C; PAWP: Post-acrosomal sheath WW domain-binding protein; NLS: Nuclear localisation signal; OAD: Oocyte activation deficiency; AOA: Artificial oocyte activation; MOAT: Mouse oocyte activation test; PIV: Particle image velocimetry.

## Misc

Walaa M Ramadan and Junaid Kashir contributed equally.

## Competing interests

The authors declare that they have no competing interests.

## Authors’ contribution

WMR, JK, CJ, and KC all contributed to the writing of this manuscript. All authors read and approved the final version of this manuscript.
